# Dispersal Beyond Mountains and Borders: Asymmetrical Gene Flow Helps Maintain Brown Bear Metapopulation Connectivity on the Balkan Peninsula

**DOI:** 10.1002/ece3.72691

**Published:** 2025-12-18

**Authors:** Charilaos Pylidis, Ivana Matic, Mihajla Djan, Kostadin Valchev, Duško Ćirović, Simeon Arangelov, Sam Walker, Naroa Sarasua, Veronika Mrazek, Ali E. Basuony, Michael W. Bruford, Koen Cuyten, Frank Hailer

**Affiliations:** ^1^ Balkani Wildlife Society Sofia Bulgaria; ^2^ Organisms and Environment, School of Biosciences Cardiff University Cardiff UK; ^3^ Faculty of Sciences University of Novi Sad Novi Sad Serbia; ^4^ Faculty of Biology University of Belgrade Belgrade Serbia; ^5^ University of the Basque Country (UPV/EHU) Leioa Spain; ^6^ Department of Zoology, Faculty of Science Al‐Azhar University Cairo Egypt; ^7^ Cardiff University‐Institute of Zoology Joint Laboratory for Biocomplexity Research (CIBR) Beijing China; ^8^ Bears in Mind Rhenen the Netherlands

## Abstract

The Balkan Peninsula is a biodiversity hotspot, harbouring some of the largest transboundary populations of large carnivores such as brown bears. However, most studies to date in this area have addressed bear population dynamics within the confines of country borders, which presents limitations. Our aim was to address this, by investigating the genetic structure and dispersal dynamics of the Eastern Balkan brown bear population and its connectivity with the Dinaric‐Pindos population from four mountain ranges and across country borders: Stara‐Planina and Rhodope in the east and Stari‐Vlah and Sharr Mountains in the west. Using both microsatellite markers and mitochondrial DNA data we detected a primary east–west cline in genetic structure, with additional north–south substructuring and high diversity overall. Evidence of past bottlenecks, historical isolation and ongoing cross‐border, and primarily male‐mediated gene flow, suggests complex dispersal dynamics shaped by past demographic fluctuations. Results from mitochondrial data further reflected signals from postglacial population contractions, re‐expansions of Balkan populations. These findings emphasise the need for conservation strategies that maintain transboundary connectivity in this metapopulation, to ensure the long‐term viability of brown bears on the Balkan Peninsula.

## Graphical abstract

We investigated the genetic structure and connectivity of the two core brown bear populations in the Balkan Peninsula. Using microsatellite and mitochondrial DNA, we found weak but clear population structure, yet also signals of functional connectivity among subpopulations preserved by asymmetrical gene flow, We discuss the implications of our findings for regional conservation strategies for the conservation brown bears in one of Europe's most biodiverse landscapes.

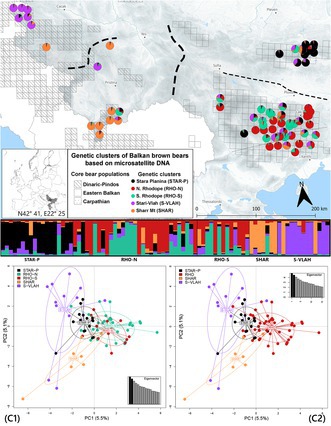

## 1 Introduction

One of the key processes for sustaining viable animal populations is functional connectivity—the ability of animals to move across landscapes and breed. At the individual level, movement is crucial for survival and reproduction. Animal movement enables access to resources (Whittington et al. 2022), avoidance competition, dispersal (Iannella et al. 2024), finding mates (Gompper et al. 1998), engaging in social interactions, avoiding humans (Stillfried et al. 2015), and responding to environmental pressures (Habib et al. 2021). At the population level, movement drives migration and gene flow and, in the long term, range shifts. Gene flow underpins this process and supports population health by reducing the risks of inbreeding depression and genetic drift (Slatkin 1987; Frankham 1996; Lacy 1987). Conversely, isolated wildlife populations can suffer from inbreeding depression, rendering them more vulnerable.

Humans are a major driver of disruption of wildlife movement population dynamics on a global scale (Larson et al. 2019; Nickel et al. 2020). Humans act as an evolutionary force, both disrupting and facilitating population connectivity (Crispo et al. 2011; Miles et al. 2019), along with global declines in genetic diversity (Shaw et al. 2025). Land use, habitat fragmentation, infrastructure development, human presence, and recreational activities (Bolas et al. 2025) impact behaviour, movement, distribution, and ultimately gene flow (Carvalho et al. 2018; Doherty et al. 2021), with the persecution of animals by humans playing a major role. This is particularly relevant in the heavily human‐modified landscapes of Europe, where species with large spatial requirements such as large carnivores coexist with humans (Chapron et al. 2014; Ingeman et al. 2022).

The Balkan Peninsula has long served as a refugium for Europe's largest terrestrial carnivore, the brown bear ( *Ursus arctos* ) (Hewitt 1999; Sommer and Benecke 2005). Once widespread across Europe and the northern hemisphere, brown bears have been extirpated from much of their historical range due to human persecution and habitat loss in the 20th century (Servheen 1990). Today, following that rapid decline, the species persists in Europe in large but fragmented populations in Scandinavia, Karelia, the Pyrenees, the Apennines, the Alps and the Balkans (Kaczensky et al. 2024). In recent years, brown bear populations in the region have shown stable or increasing trends, aided by favourable conservation policies, rural depopulation, reforestation and transboundary projects (Reinhardt et al. 2019; Cimatti et al. 2021; Kaczensky et al. 2024).

In the Balkans, bear populations are commonly grouped into the larger Dinaric‐Pindos in the west and the Eastern Balkan population, which differs in size and fragmentation levels (Kaczensky et al. 2024). The Eastern Balkan population of brown bears spanning Bulgaria and Greece occupies a unique and ecologically important position at the southern edge of its European range. To the north is the Stara Planina Mt. range (Central Balkan Mountains), while to the south, the larger Rilo‐Rhodopean massif—situated at the ecological crossroads of continental Europe, Asia, and the Mediterranean basin—extends across southern Bulgaria and NE Greece. The larger subpopulation—located in the Bulgarian part of the distribution—was recently estimated to be below the threshold of 500 adult individuals (Frosch et al. 2014), which is considered the minimum for the long‐term viability of a population (Allendorf et al. 2012). It is thought that the population was at its lowest level in the 1930s, with its recovery impeded by legal hunting in the 1970s and 1980s and poaching since the 1990s when it was assigned protected status (Dutsov et al. 2008). In southern Rhodope in Greece, the bear population was estimated at around 90 individuals (Pylidis et al. 2021). Movement of bears in the region is not restricted by the border between the two countries, allowing individuals to roam freely across the continuous habitat.

The significant population size reductions due to historical persecution raise concerns about the genetic connectivity between population segments and metapopulation viability in the Balkans. With current extensive subdivision by political borders, the Balkans represent a vast transboundary complex of habitats and management regimes. To date, much brown bear research in the region has focused on units defined by national or regional borders, providing useful baseline genetic diversity data (summarised in Skrbinšek et al. 2012). In some cases, studies have taken an even more localised approach (e.g., Karamanlidis et al. 2012; Karamanlidis, Pllaha, et al. 2014; Karamanlidis, Stojanov, et al. 2014; Tsaparis et al. 2014; Tsalazidou‐Founta et al. 2024), which limits the ability to evaluate populations on a broader scale. Conservation strategies increasingly emphasise the need for genetic monitoring and the maintenance or re‐establishment of connectivity between populations to safeguard adaptive diversity (Thomas et al. 2022, 2025; Bogdanović et al. 2023). The long‐term viability of metapopulations depends on their functional connectivity across landscapes, as gene flow between population fragments increases their resilience. In the Balkans, where various emerging anthropogenic phenomena such as infrastructure development and climate change threaten habitats and populations, evaluating the functional connectivity for bears and other large carnivores at a population level is becoming a priority (Linnell et al. 2008; Tammeleht et al. 2010; Matosiuk et al. 2019).

In this context, we set out to investigate the population genetics of bears in the core areas of their Eastern Balkan distribution. Although evidence of gene flow with the Dinaric‐Pindos has been reported at the southern margin of its European range (Pylidis et al. 2021), the functional connectivity within the core of its western Balkan distribution—and especially across complex transboundary landscapes—remains unknown. This region is a focal point for understanding postglacial recolonization dynamics, population structure and conservation genetics, and represents a contact zone between the Eastern and Western mtDNA lineages of brown bears (Frosch et al. 2014; Pylidis et al. 2021). Utilising both biparentally inherited microsatellite loci and maternally inherited mitochondrial DNA, we examined patterns of genetic variability, population structuring and gene flow. This approach facilitates an understanding of population dynamics in recent and ancient periods and yields significant recommendations for the conservation and management of populations, such as the definition of management units (Waits et al. 2001; Šnjegota et al. 2021).

## 2 Materials and Methods

### 2.1 Data Collection and Sample Grouping

**FIGURE 1:**
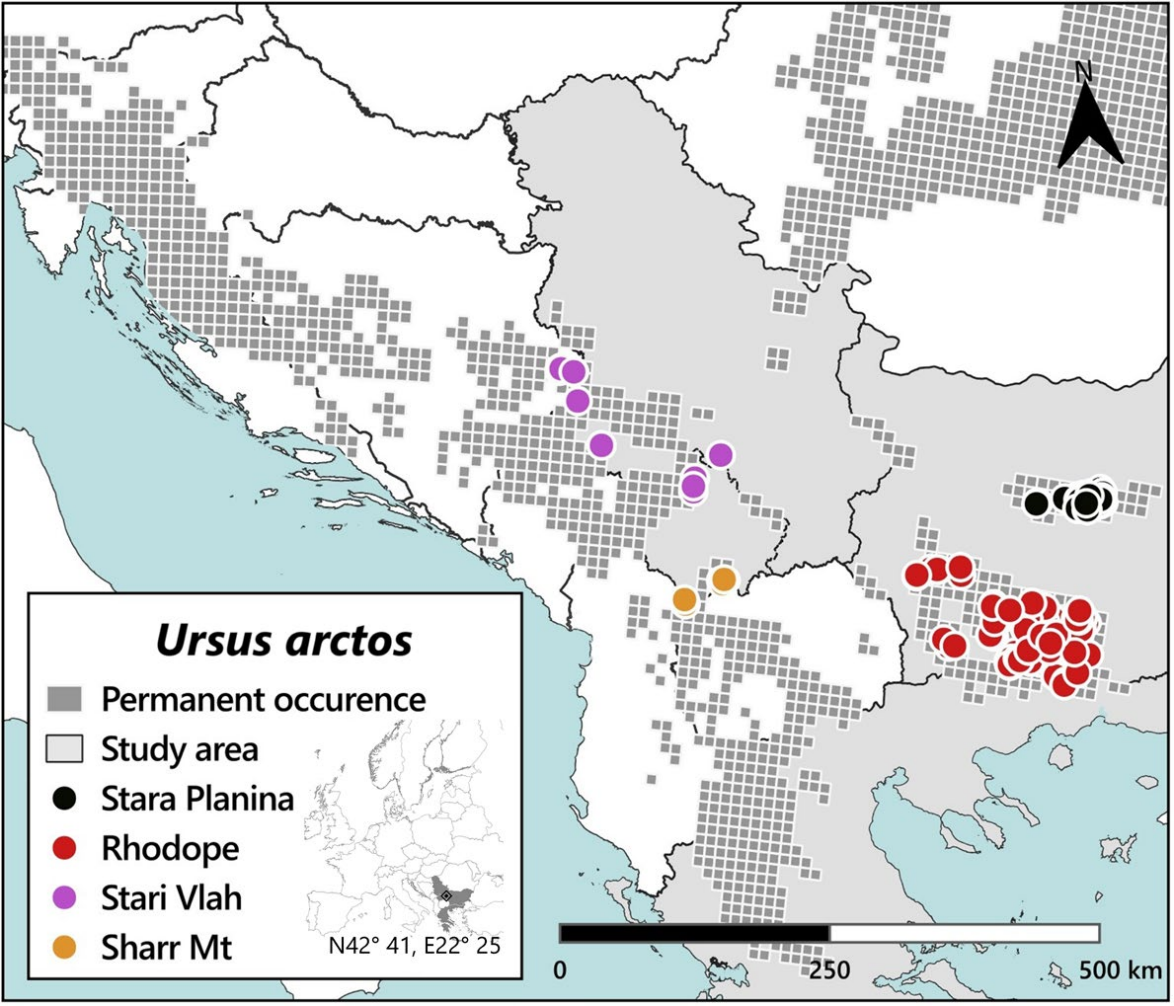
Sampling locations over a grid for the permanent brown bear occurrence in the Balkan peninsula (*Data Source:* Kaczensky et al. 2013). Colours (black, red, purple and orange) represent bear populations from Stara Planina and Rhodope of the Eastern Balkan population, and Stari Vlah and Sharr Mountains of the Dinaric–Pindos.

We sampled the eastern distribution of the Balkans, focusing on the core area of bear distribution in Stara Planina (Balkan Mountains) and the Rilo‐Rhodopean massif across both sides of the border between Bulgaria and Greece. For a broader comparison with the Dinaric‐Pindos distribution, we obtained samples from the Stari‐Vlah region of the High Dinaric Alps (N 43°55′, E19°21′ including the Kopaonik and Mokra mountains further south) and the northern tip of the Sharr Mountains (N 42°09′, E 20°47′ of the Scardo‐Pindic mountain system) (Figure 1). We grouped the samples by mountain ranges rather than by country, resulting in four groups, corresponding to the following mountain ranges: Stara Planina (STAR‐P) and Rhodope (RHO) in the Eastern Balkan distribution, and Stari‐Vlah (S‐VLAH) and (SHAR) in the Dinaric‐Pindos range. Our sampling effort was skewed in favour of the EB population, as we had been sampling more intensively for a population‐estimation study focused on that area. All analysed samples were scats, apart from three hair samples and 10 blood samples that were sampled ethically from DIN‐PIN bears. To avoid issues with low sample sizes, for certain analyses (e.g., effective population size, bottleneck detection and Bayesian migration estimation), when biologically justified, we merged the two western population groups (DIN‐PIN and SHAR) into a single unit.

### 2.2 Sample Screening and Microsatellite Amplification

Stool and hair samples were extracted in a laboratory free of PCR products and dedicated to the analysis of low copy DNA samples, using Qiagen QIAamp Fast DNA Stool Mini Kits, with a modification in the suspension stage increased to 95°C if the sample did not lyse. For improved eluting efficiency, the elution buffer was warmed up to 60°C for 10 min before flushing the buffer twice through the spin column. DNA extraction for the blood samples was performed using an ammonium acetate salting‐out approach, followed by ethanol precipitation and resuspension (Sambrook and Russell 2001). Initially, all DNA extracts were screened for amplification using a mitochondrial DNA (mtDNA) fragment (see section below), but as successful mtDNA amplification did not guarantee microsatellite amplification, we switched to screening samples with microsatellites.

We initially tested a panel of 12 microsatellite loci amplified in two multiplex PCR reactions, but dropped two loci (Mu10 and G10B) due to amplification issues, leaving us with two final multiplex sets encompassing 10 loci (Table 1): G10J, Mu51, Mu05, G10L, G1A (multiplex 1), and Mu50, Mu59, G10C, Mu23, G10P (multiplex 2) (see Data S1). These markers are commonly used in bear population genetics due to their high amplification success and polymorphism levels. Samples that gave clear results for at least 2 loci per multiplex from one PCR replicate were then amplified further. Any sample that failed was discarded, and the remaining samples were typed an additional 2 to 7 times. As reference samples, we used DNA from bear tissue samples. We scored the genotypes manually and proceeded to create consensus genotypes. We used the match function in GenAlEx 6.5 (Peakall and Smouse 2012), allowing 6 mismatches including missing loci. Alleles were accepted if they appeared at least two times. The only individuals represented by a single profile, were genotypes derived from blood samples. Mismatch ambiguities that could not be verified by rechecking the scoring were discarded. We then pooled the matching genotypes and used them for all downstream analyses.

**TABLE 1:** Estimates of autosomal genetic diversity for 82 Pindo‐Dinaric and Eastern Balkan bears (dataset without close relatives) based on 10 microsatellite loci from 4 mountain ranges.

Pop	*n*	MNA	*N*_Eff_	*A*_*R*_	PA	*H*_o_	*H*_e_	*F*_is_
STAR‐P	18	5.7	3.78	3.6 (0.60)	0	0.594 (0.047)	0.732 (0.032)	0.165
RHO	44	7.8	4.17	3.8 (0.50)	0.04 (0.03)	0.636 (0.034)	0.736 (0.027)	0.136
SHAR	7	4.8	3.67	3.7 (1.00)	0.07 (0.07)	0.802 (0.056)	0.744 (0.041)	−0.163
S‐VLAH	13	6.3	4.26	4.0 (0.50)	0.06 (0.02)	0.700 (0.051)	0.784 (0.018)	0.074

*Note:* Values in brackets denote standard errors.

Abbreviations: *F*_is_, inbreeding coefficient; *H*_e_, mean unbiased expected heterozygosity; *H*_o_, mean observed heterozygosity; MNA, mean number of alleles per locus; *n*, number of individuals for each grouping; *N*_Eff_, effective number of alleles per locus; PA, private alleles.

We estimated PID, the probability of identifying identical genotypes, for randomly chosen unrelated individuals, and PID_SIB_, the probability of identifying siblings with the same genotypes, as a function of the number of loci used in the study (Waits et al. 2001), and used the information to estimate the minimum number of loci that could be used to allow distinguishing between genotypes in our dataset. Match analysis of 151 samples resulted in 122 unique genotypes, that is, individuals. Hence, our final dataset contained 122 multilocus microsatellite genotypes, each with a minimum of 7 genotyped loci, with the following sample sizes per region: STAR‐P, *n* = 72; RHO, *n* = 158; S‐VLAH, SHAR, *n* = 8. Sexing was done using the double‐Y method (Bidon et al. 2013) (see Data S3) on a subset of samples, due to limited resources and DNA extract remaining after microsatellite genotyping and mtDNA sequencing.

### 2.3 Locus and Dataset Assessment

GenAlEx 6.5 (Peakall and Smouse 2012) was used to calculate observed and unbiased expected heterozygosity, numbers of alleles per locus, Weir and Cockerham's (1984) index of population differentiation (*F*_ST_), and for the test of deviation from Hardy–Weinberg equilibrium (HWE). Allelic richness was calculated using a rarefaction procedure implemented in the package Hierfstat (Goudet 2005) in R 4.5.0 (R Core Team 2025). There was a lack of significant deviation from HWE across all four geographical populations for all loci, and all 10 loci were used for subsequent analyses. We then proceeded to remove individuals which had ≥ 0.3 pairwise relatedness to another individual in the dataset, determined using ML‐RELATE (Kalinowski and Taper 2006), as the effect of relatives inflates genetic structure and alters allelic frequencies, impacting a variety of downstream analyses. Once these close relatives were removed, the final dataset contained 82 microsatellite profiles.

### 2.4 Population Structuring, Gene Flow and Effective Population Size

We used STRUCTURE 2.3.4 (Pritchard et al. 2000) to infer cluster membership proportions, using (i) the whole dataset (*n* = 122), and (ii) the dataset excluding close relatives (*n* = 82), (iii) for bears from all mountain ranges (*n* = 96), and (iv) for EB bears only (*n* = 62), based on the admixture model and assuming correlated allele frequencies, but without location data as a prior. We divided the Rhodope dataset into two geographical subgroups—northern (RHO‐N) and southern (RHO‐S)—to determine whether any differentiation followed a north–south geographical pattern across the border and to assess the level of potential gene flow exchange. All STRUCTURE analyses were performed with 500,000 MCMC replicates, a burn‐in of 250,000, and with 10 independent runs for each value of K ranging from 1 to 10. Results were summarised and visualised using STRUCTURE SELECTOR (Li and Liu 2018).

Unidirectional gene flow estimates between sampling locations were obtained by Bayesian analysis in BAYESASS (Wilson and Rannala 2003), based on multiple runs with independent starting ‘seeds’, and following the software manual to achieve adequate mixing of MCMC chains (acceptance rates being between 30% and 60%) and chain convergence (inspection of trace plots in R), eventually choosing a burn‐in of 50 million among a total chain of 500 million steps. For result reporting we only used reliable MCMC outcomes, as determined by the Bayesian script DEVIANCE (Meirmans 2014).

To test the presence of isolation‐by‐distance (IBD; Wright 1943), we carried out spatial autocorrelation analysis, equivalent to stratified Mantel tests, in SPAGeDi 1.2 (Hardy and Vekemans 2002) using distance classes of 20 km. The significant association between genetic and geographical distance within the EB dataset and in bears from all geographical populations was checked using the kinship coefficient (*F*_*ij*_; Loiselle et al. 1995) as a pairwise estimator of relatedness in 10,000 permutations. We used BARRIER v2.2 to visualise linear *F*_ST_ on the landscape (Manni et al. 2004).

For the detection of the first‐generation migrants, we estimated the likelihood ratio *L_home/L_max* in GeneClass (Piry et al. 2004) using a Bayesian method for computation (Rannala and Mountain 1997) in 10,000 permutations and the simulation algorithm (Paetkau et al. 2004) with a threshold *p*‐value of 0.01.

Effective population size was calculated only for our focus area of the Eastern Balkan population in LDNe (Waples and Do 2008). We looked for signatures of a genetic bottleneck using the Infinite Allele Model (IAM) and Two‐Phase Model (TPM) in BOTTLENECK (Piry et al. 1999), exploring different rates of stepwise mutation for the TPM. In addition, to identify allele losses that may serve as indicators of recent population bottleneck events, we calculated the Garza‐Williamson (G‐W) statistic (Garza and Williamson 2001).

### 2.5 Mitochondrial DNA Control Region Sequencing and Data Analysis

We amplified a portion of the maternally inherited mitochondrial DNA (mtDNA) control region. Samples were initially tested using the primer pair URS1_F (5′‐ACAGCTCCACTACCAGCACCC‐3′) (Hailer et al. 2012) and UCRS_R2 (5′‐CGTTCGTTCGATTTAGTGGCG‐3′; newly designed as part of this study), which amplifies a 671 bp fragment including the primers. In addition, a second primer pair, CR_F (5′‐AGGAAGAAGCAACAGCTCCACTA‐3′) and CR_R (5′‐CCATCGAGATGTCCCATTTGAAG‐3′) (Matosiuk et al. 2019), was used if the longer first fragment failed to amplify. This second primer pair yielded a 431–434 bp fragment including primers. Details of amplification conditions are provided in Data S2. The final dataset comprised 96 newly obtained sequences that were used for further analyses, along with reference data from Genbank (Appendix S2).

Molecular diversity indices, including the number of polymorphic sites, haplotype diversity (*H*_d_), nucleotide diversity (*π*) and the mean number of pairwise differences (*k*), were calculated using DnaSP v5.10 (Librado and Rozas 2009). Parameters of molecular diversity were also calculated separately for each of the four population groups. Genetic differentiation among populations was evaluated in Arlequin (Excoffier and Lischer 2010) through analysis of molecular variance (AMOVA). To assess demographic history, mismatch distribution analysis based on pairwise differences was performed in Arlequin to test for deviations from the sudden expansion model. Neutrality tests were performed using Tajima's *D* (Tajima 1989) and Fu's *F*s (Fu 1997) in Arlequin, and Fu and Li's *F* and *D* statistics in DnaSP. Phylogenetic relationships among identified haplotypes were inferred by constructing a median‐joining (MJ) network (Bandelt et al. 1999) in PopART (available at http://popart.otago.ac.nz). To assess these relationships in a broader phylogenetic context, we also included sequences from public databases, resulting in a total dataset of 153 sequences. Spatial patterns of molecular diversity based on nucleotide variability in the mitochondrial control region were assessed using the Geneland v.4.0.5 package (Guillot et al. 2005) in R. The analysis was conducted under the uncorrelated model, with 10 independent MCMC runs of 1,000,000 iterations each, performed separately for each value of *K* (ranging from 2 to 10).

## 3 Results

### 3.1 Microsatellite Variability: Genetic Structure and Functional Connectivity

Cumulative *PID* for the 10 loci was 6.2 × 10^−12^ and *PIDsib* was 6.8 × 10^−5^. The analysis showed that 5 loci could be used for effective individual identification with 0.01 confidence, but to be conservative we used a minimum of 7 loci, which corresponded to 0.001 confidence for the identification of siblings (see Data S4; full genotype data provided in Appendix S2).

All loci were found to be polymorphic in all populations, with the average number of alleles ranging from 4.8 to 7.4 (Table 1). Considering the different sample sizes representing four geographical populations, expected heterozygosity gives a more valid insight into genetic diversity across the populations of our dataset. Expected heterozygosity across the 10 loci ranged from 0.732 for SHAR in the west to 0.784 for S‐VLAH in the east (Table 1). The largest number of private alleles was detected in Rhodope (10 private alleles over eight loci), and three private alleles over three loci were detected in bears from the S‐VLAH population. Linkage disequilibrium was significant in 8.8% of the pairwise comparisons across all populations, likely reflecting Wahlund effects and admixture. Pairwise *F*_ST_ values for all populations were low to moderate, ranging from 0.034 to around 0.079—with the SHAR population found to be most distinct, followed by STAR‐P and the lowest between the two clusters seen in Rhodope (Table 2).

**TABLE 2:** Pairwise genetic differentiation between sample groupings of Pindo‐Dinaric and Eastern Balkan bears.

Population	Eastern Balkan		Dinaric‐Pindos	
	STAR‐P	RHO	SHAR	S‐VLAH
STAR‐P	—	0.034	0.079	0.060
RHO	0.065	—	0.070	0.060
SHAR	0.723	0.502	—	0.076
S‐VLAH	0.452	0.291	0.476	—

*Note:* Above diagonal: *F*_ST_ based on microsatellite loci; below diagonal: *Φ*_ST_ based on mtDNA.

**FIGURE 2:**
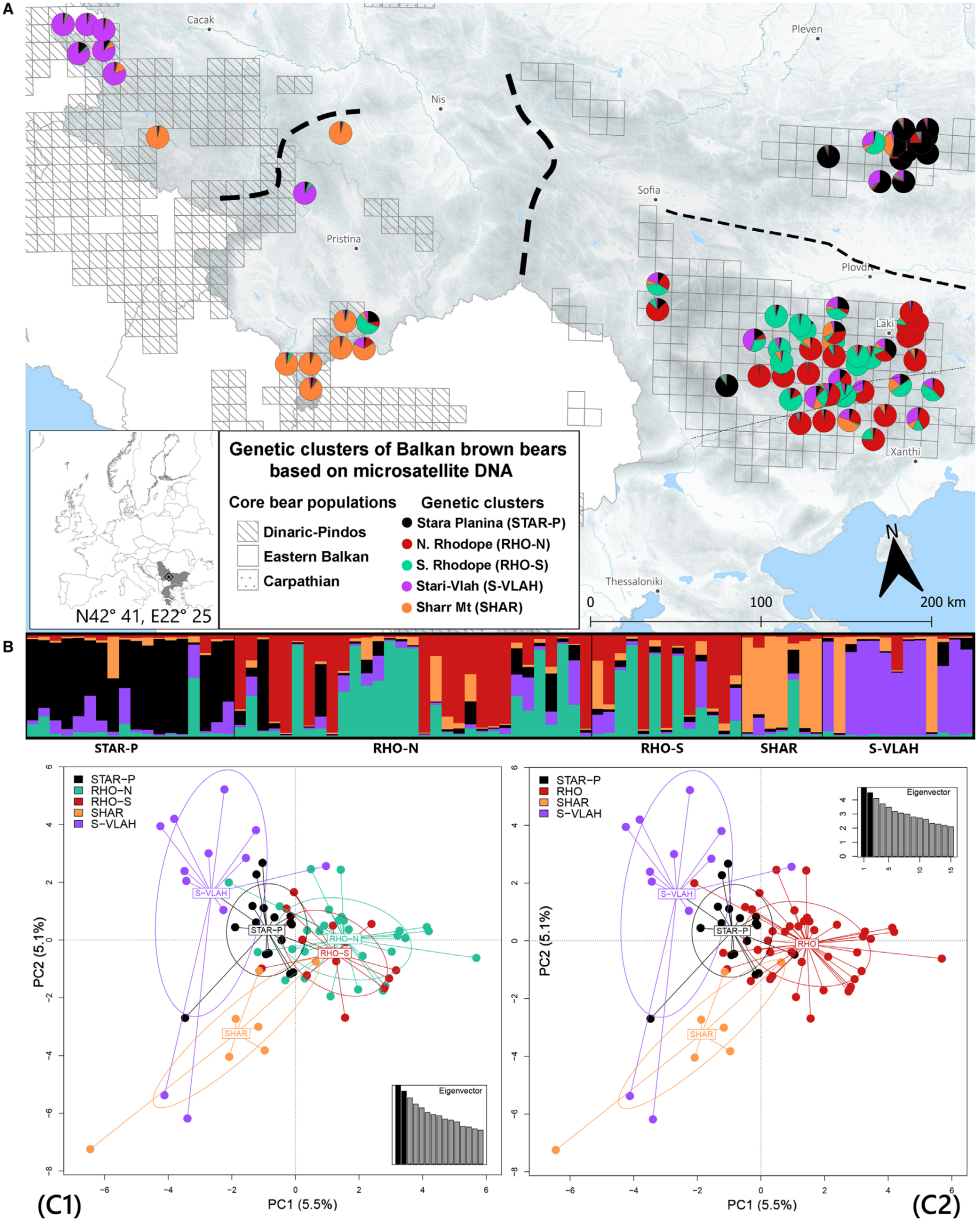
Genetic structuring for microsatellites. (A) Genetic clusters of 82 Eastern Balkan and Dinaric‐Pindos brown bears' genotypes, indicated by STRUCTURE, with landscape‐level differentiation (dashed lines) indicated by BARRIER v2.2, line width corresponding to the strength of the barrier based on linear *F*_ST_. (B) Corresponding bar plot from STRUCTURE. (C1, C2) Principal coordinate analysis (PCA) plots for 5 and 4 groups, respectively. Inertia ellipses encompass 1.28 times the standard deviation.

**FIGURE 3:**
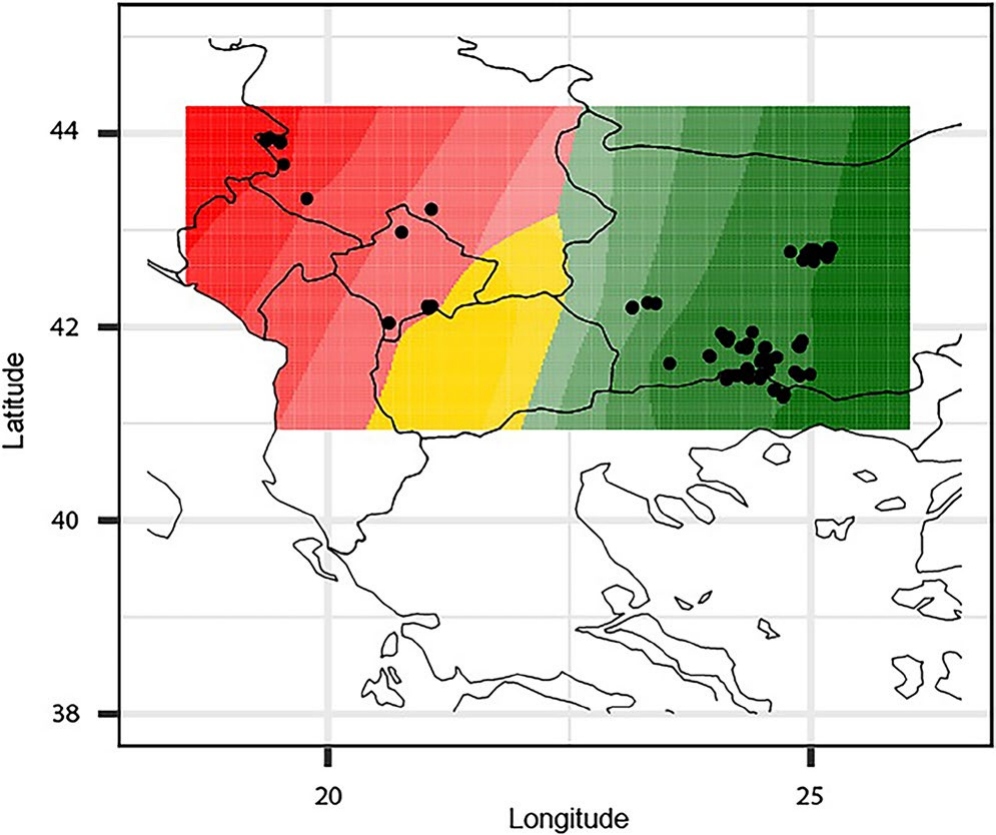
Spatial genetic structure of brown bears based on mtDNA control region sequences, inferred from GENELAND analysis. Posterior probability distribution for cluster membership of individual bears is shown on the map. Regions with darker shading correspond to the highest probabilities of cluster membership. Different clusters are represented by red, yellow and green colors. Geographical locations of individuals are indicated by black dots.

Bayesian analysis in STRUCTURE with all individuals from all populations (dataset without relatives; *n* = 82) suggests a clear but weak genetic clustering with Δ*K* and LnP(*K*) indicating *K* = 5 as the most likely scenario (Data S5), with a subdivision of bears from Rhodope into two clusters (Figure 2). When we run the dataset without relatives for the Eastern distribution only, Δ*K* and LnP(*K*) indicated *K* = 3 with a similar subdivision for Rhodopean (Data S5.2). Running the analysis with all individuals present in the dataset created an additional cluster in both cases (Data S5.3 and S5.4). This cluster always appeared in Rhodope while the overall clustering pattern remained unchanged for all other populations. The substantial overlap between the two Rhodope clusters was indicated when we run PCA with 5 clusters (Figure 3), so we decided not to partition the Rhodope into two groups for all other analyses. The genetic clusters were found to correspond to the four main geographical populations. The results show a strong east–west structuring between EB and DIN‐PIN with additional north–south substructuring within the two main Balkan populations. Based on a *Q*‐value threshold of 0.75, 15.9% of individuals were assigned to cluster 1, 18.3% to the second cluster, 12.2% to cluster 3, 8.5% to cluster 4, 11% to cluster 5, with 34% of the individuals classified as admixed, which was especially pronounced in Rhodope, while the other three populations were more distinct (Figure 3). PCA also revealed a pronounced east–west differentiation between all groups and a secondary north–south gradient within the two main Balkan populations.

Spatial autocorrelation results showed there is a clear spatial genetic structure at short distances (≤ 21 km), where individuals are significantly more genetically similar than expected under random mating. At larger distances, the spatial genetic structure fades, with no consistent autocorrelation, suggesting gene flow or random mating dominates at these scales.

Estimates of recent migration from BAYESASS (see Data S6) revealed asymmetrical gene flow and low recent migration. The highest was from RHO to SHAR (8.84%), being highly asymmetrical (0.5% in the opposite direction). Local ancestry of individuals ranged from 89.0% for S‐VLAH to 94.6% for RHO. GENECLASS detected eight first‐generation migrants; three of whom were also assigned as migrants by STRUCTURE. All these three were individuals inferred to have moved between EB and DIN‐PIN (Table 3).

**TABLE 3:** First generation migrants and directional movement detected by assignment tests in GENECLASS.

Migration direction	Origin	Migration to	−log(L)	Sex
Within Eastern Balkan	Rhodope	Stara Planina	9.133 (0.0067)	Unidentified
	Rhodope	Stara Planina	11.501 (0.0000)	Unidentified
	Stara Planina	Rhodope	13.324 (0.0042)	Male*
Within Dinaric‐Pindos	Sharr Mt	Stari‐Vlah	11.643 (0.0000)	Likely male*
	Sharr Mt	Stari‐Vlah	14.386 (0.0035)	Male*
Eastern Balkan → Dinaric‐Pindos	Stari‐Vlah	Stara Planina	11.372 (0.0098)	Unidentified
	Sharr Mt	Rhodope	14.202 (0.0006)	Likely male
Dinaric‐Pindos → Eastern Balkan	Sharr Mt	Rhodope	12.047 (0.0004)	Male

*Note:* Individuals marked with an asterisk indicate migrants detected by STRUCTURE, based on a *Q* value threshold of 0.75.

### 3.2 Effective Population Size and Bottleneck Signals

Effective population size inferred by LDNE for the Eastern Balkan population groupings (Table 4) was found to vary between 77.8 (58.4–109.8) and 82.2 (63.3–112.1) depending on the frequency threshold. When computed for each of the two EB subpopulations, it was the highest for RHO and lowest for STAR‐P (with a much wider confidence interval, likely a function of its smaller sample size). Evidence of a bottleneck in all groups under the IAM model while for Dinaric bears bottleneck signal was detected for three out of four TPM scenarios (see Data S7.1). The G‐W statistic values for all four populations were found to be below 0.5 (Data S7.2). All allele frequency distributions were found to be L‐shaped (data not shown).

**TABLE 4:** Effective population size (*N*_e_) and 95% CI based on a linkage disequilibrium approach for the Eastern Balkan population of brown bears.

Population	*N*	Frequency threshold		
		0.05	0.02	0.01
STAR‐P	27	34.7 (17.7–130.3)	42.9 (24.0–122.7)	46.1 (24.6–162.6)
RHO	69	77.2 (53.0–127.5)	77.6 (59.1–108.1)	68.7 (49.6–103.4)
Total	96	77.8 (58.4–109.8)	82.2 (63.3–112.1)	79.7 (59.4–113.5)

*Note:* Results are shown for each of the geographical populations, separately for three different frequency thresholds (0.05, 0.20, 0.01).

### 3.3 Mitochondrial DNA Results

Genetic diversity parameters and AMOVA were conducted based on populations grouping revealed by microsatellites and which correspond to their geographical origin. In the dataset of 96 sequences newly generated in our study, we detected 17 haplotypes, based on 34 polymorphic sites (Table 5). When including additional data from Genbank, the dataset included 153 sequences, comprising 26 distinct haplotypes (Appendix S2).

**TABLE 5:** Molecular diversity indices based on mtDNA control region sequences.

Molecular diversity	STAR‐P	RHO	S‐VLAH	SHAR	Total
Number of sequences (*n*)	24	49	17	6	96
Number of haplotypes (*h*)	4	9	6	1	17
Number of polymorphic sites (*S*)	25	29	13	—	34
Haplotype diversity (*H*_d_)	0.370	0.672	0.706	—	0.740
Nucleotide diversity (*π*)	0.008	0.023	0.013	—	0.019
Average number of pairwise differences (*k*)	2.627	7.854	4.294	—	6.285
Mismatch distribution and neutrality test					
Sum of squared deviations (SSD)	0.0780	0.080	0.090		0.119
Tajima's *D*	−2.260**	0.690	0.442	—	−0.156
Fu's *F*s	3.068	5.626	1.638	—	0.683
Fu and Li's *F*	−3.690*	1.266	0.469	—	0.511
Fu and Li's *D*	−3.551*	1.269	0.393	—	0.803

*Note:* Results shown are for four brown bear groups, including variability, neutrality and mismatch distribution statistics.

* *p* < 0.05.

** *p* < 0.01.

Molecular diversity analyses based on mtDNA control region revealed notable variation in genetic diversity across the studied populations. The STAR‐P population exhibited the lowest haplotype diversity (*H*_d_ = 0.370) and nucleotide diversity (*π* = 0.008), whereas RHO and S‐VLAH populations showed higher genetic diversity, with haplotype diversities of 0.672 and 0.706, and nucleotide diversities of 0.023 and 0.013, respectively. SHAR was represented by one single haplotype, preventing further insight into its genetic diversity.

The highest number of haplotypes was found in the RHO population (9), while only one haplotype was detected in all six individuals from the SHAR population. Mismatch distribution analysis did not show a statistically significant deviation from expectations under a sudden expansion model in the total dataset, nor in any of the populations containing at least two haplotypes. Tajima's *D* test showed a statistically significant negative value in the STAR‐P population. The same was revealed by Fu and Li's *F* and Fu and Li's *D* tests; however, values were positive for other populations and the whole dataset.

We employed GENELAND analyses to gain insight into the distribution of genetic variation across the landscape. The results show east–west differentiation, corresponding to DIN‐PIN and EB populations (Figure 3). An additional cluster has been detected comprising individuals from SHAR, with lower support (~0.4).

**FIGURE 4:**
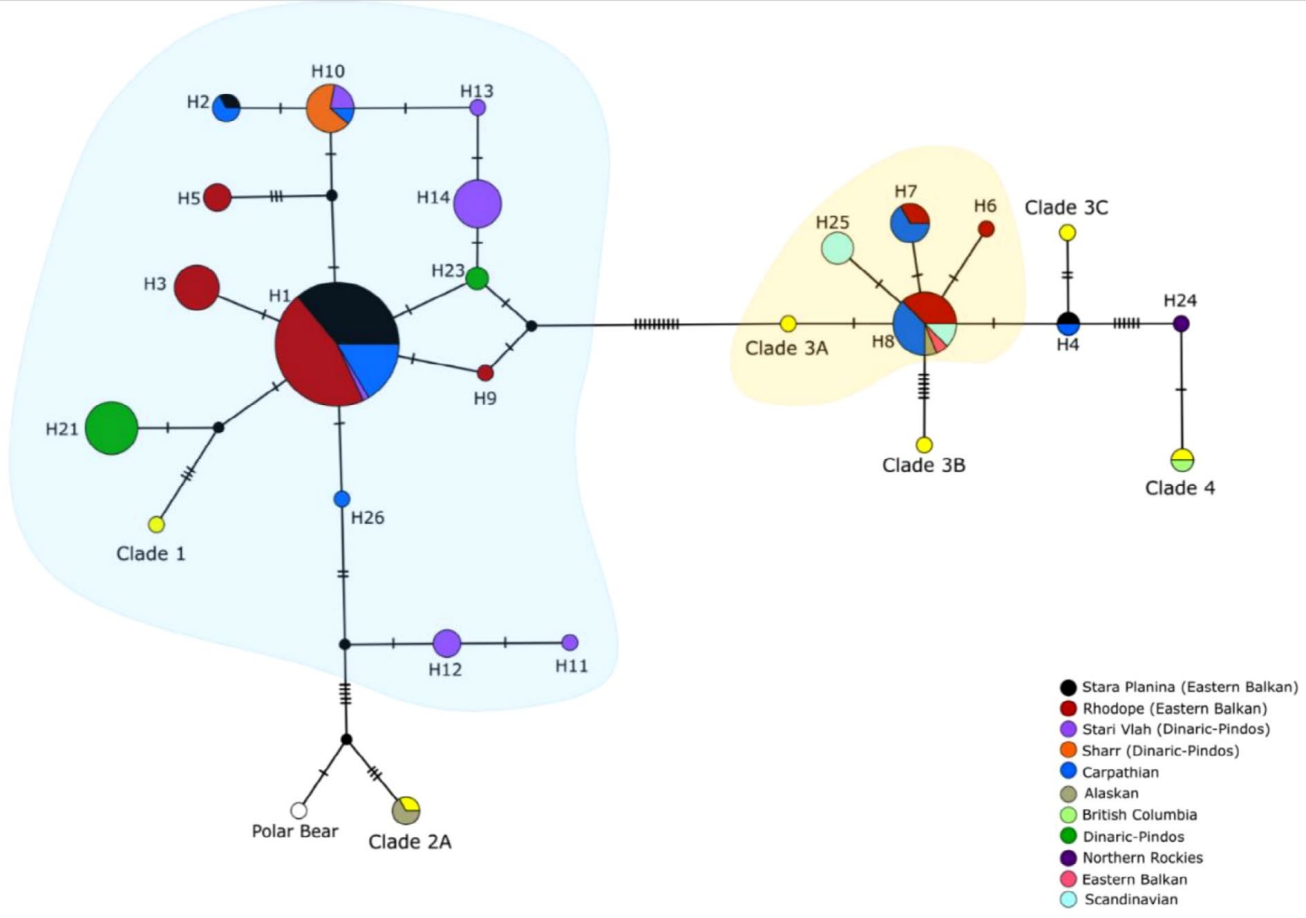
Median‐joining network of the mtDNA control region haplotypes based on a dataset comprising 153 sequences. In addition to 96 sequences generated in this study, 57 sequences were retrieved from public databases, including representative haplotypes from six distinct clades of bear populations. Haplotypes belonging to Clade 1 are shown with blue background shading, and those belonging to Clade 3A with yellow background shading, denoting the two major haplogroups identified in this dataset. Additional brown bear clades not encountered in the study area are shown as yellow circles, plus the North American haplotype H24 (Appendix S2).

AMOVA showed a significant percentage of variation among populations (32.21%), and within populations 67.79%. Pairwise *Φ*_ST_ based on mtDNA sequences (Table 2) both the lowest genetic difference between STAR‐P and RHO populations. The highest *Φ*_ST_ was found between STAR‐P and SHAR. Haplotype grouping corresponded mostly with EB and DINPIN populations. Moreover, the most abundant haplotype in our dataset (H1) was present across three out of four geographical populations. H1 was most frequent in EB, and both EB and DIN‐PIN showed the presence of private haplotypes (Figure 4).

## 4 Discussion

Here we present the first study of the Eastern Balkan (EB) population of brown bears and an assessment of its functional connectivity with the Dinaric‐Pindos population (DIN‐PIN) across country borders. Our analyses reveal a primary east–west structuring based on two types of genetic markers. Further, we detected north–south substructuring based on microsatellites only. Detection of recent long‐distance gene flow highlights the potential for a metapopulation structure persisting to this day, despite habitat fragmentation and the presence of national borders.

### 4.1 Genetic Structuring of Balkan Brown Bears

We infer that the EB population comprises two main genetic clusters: (i) one in the north, corresponding to the Stara Planina region, (ii) another in the south, aligned with the Rilo‐Rhodopean Massif. In the west, we identified two clusters: (i) one comprising bears from the Stari‐Vlah region in the High Dinaric Alps, including Kopaonik and Mokra‐Gora Mt., and (ii) another in the south consisting of bears from Sharr Mt. at the northern tip of the Scardo‐Pindic range. The three metrics we employed in microsatellite analyses—Δ*K*, LnP(*K*), and MedK—did not always converge on the exact number of populations, but the identified clusters were biologically meaningful and aligned well with known populations and sampling locations. Further, our results echo the findings of Frosch et al. (2014), who detected similar substructure of EB north of the border with Greece, reporting a clear north–south differentiation between bears from Stara‐Planina and those from Rhodope. Nevertheless, our finding of overall relatively weak but clear genetic structuring across the study area, along with evidence of functional dispersal (see below), supports the presence of a brown bear metapopulation across much of the Balkans.

Although the observed genetic structure across the Balkans is not very strong, it is consistent with regional studies on bears and large mammals in Europe (Tammeleht et al. 2010; Czarnomska et al. 2013; Šnjegota et al. 2021; Niedziałkowska et al. 2024). Overall, relatively weak population genetic structuring is expected in brown bears, due to their capability of dispersal over large distances, and range‐wide signals of male‐biased gene flow (Bidon et al. 2014). Furthermore the bear population in the Balkans has been fragmented for a large period of its recent history (Servheen 1990). In small and partially isolated populations, genetic drift intensifies, reducing genetic variation within these fragments and increasing genetic differentiation (Willi et al. 2007). As bear populations recover and those population fragments start coming into contact, the genetic signatures become less pronounced. A similar pattern has been recently observed in Yellowstone bison (Stroupe et al. 2025). This hypothesis could explain the observed and much weaker further subdivision within the Rhodopean bears into two clusters. The lack of a clear geographic pattern suggests that there is one cluster, the Rhodope population, consistent with the conclusions of Frosch et al. (2014) and the findings of Pylidis et al. (2021), who reported one genetic cluster in the Rhodope south of the border. Similarly, PCA analysis depicts the substantial overlap between the two tentative Rhodope clusters while the visualisation from BARRIER software, although heuristic, provides a clear indication of the low level of genetic differentiation (Figure 2). It is possible that a subdivision existed in the past and that we are observing the results of extensive interbreeding between the clusters, and their fusion into one panmictic population in the Rhodope alongside the border. The presence of a few close relatives in our dataset produced an additional cluster, which was always in the Rhodope population. This additional cluster was observed both when conducting the analysis with the EB bears only (Data S5.3) and with the whole dataset (Data S5.4). These findings strongly support the removal of relatives when conducting STRUCTURE analyses on populations with small effective population sizes (*N*_e_), which likely include family groups, in order not to overestimate the genetic structure.

The primarily east–west oriented structuring we observed adds further evidence to a pattern observed in other large carnivores and other taxa in the Balkans, including humans (Djan et al. 2014; Glasnović et al. 2018; Šnjegota et al. 2018, 2021; Pylidis et al. 2021; Sotiropoulos et al. 2007; Ursenbacher et al. 2008; Rezić et al. 2022; Veličković et al. 2015; Kovacevic et al. 2014). A meta‐analysis for many taxa found that eastern parts of the Balkans served as refugia for many taxa and subsequent post‐glacial expansion created east–west gradients in genetic diversity; however, the authors reported homogeneous distribution in mitochondrial diversity for brown bears (García‐Rodríguez et al. 2024). In wolves, climate variations and environmental altitude have been suggested as factors for that east–west cline (see discussion by Šnjegota et al. 2021), but ecological factors are less likely to play a role in population structure of brown bears (after Tammeleht et al. 2010). We can only hypothesise that the drivers of structure are the anthropogenic causes such as the historical fragmentation of bear populations coupled with the topography of the Balkans. Mountains act as natural barriers in bears and other large carnivores (May et al. 2008; Korsten et al. 2009), and habitat fragmentation has been shown to increase female philopatry in models (Henry et al. 2016). These factors may have been limiting animal movement and restricting connectivity between populations. As gene flow is facilitated by connected habitats that enable the movement and interbreeding of individuals from different populations (Sharma et al. 2013; Modi et al. 2025), future studies integrating movement ecology and genetic analyses could provide valuable insights that can be used for delineating the causes of genetic structure in the region, including infrastructure and the identification of corridors, which would favour not only brown bears but also other species and habitats.

### 4.2 Sex Bias in Gene Flow Patterns, and Functional Connectivity

Overall, we found higher mitochondrial than nuclear differentiation among populations, consistent with differences in inheritance, effective population size and dispersal among marker types (Hedrick 2005). While all these factors are likely to play a role for brown bears (Lawson Handley et al. 2006; Bidon et al. 2014), the contrast between low nuclear and high mtDNA differentiation is likely, in part, due to male‐biased gene flow, where males move between populations more frequently (and/or further) than females. Mitochondrial DNA data reveal moderate to very high genetic differentiation, indicating stronger matrilineal structure, as described previously for brown bears (e.g., Keis et al. 2013; Bidon et al. 2014). Further, geographic features such as mountain ranges and human‐dominated lowlands can further restrict their movement (García‐Sánchez et al. 2022). These physical barriers can lead to genetic divergence even between populations in neighbouring areas, despite the species' overall ability to disperse over long distances. In southern Scandinavia, a low‐density bear population zone has been suggested to limit female dispersal, maintaining mtDNA population structuring (Taberlet and Bouvet 1994). Similarly, de Jong et al. (2023) described how geographic features may restrict female movement and maintain matrilineal structuring, despite geographic proximity.

Our evidence for functional connectivity both in the eastern population and between EB and DIN‐PIN is strong. Overall, GENELAND detected eight likely first‐generation migrants, three of which were also detected by STRUCTURE. While it was only possible to determine the sex with certainty in three out of these eight individuals, with the sex of three individuals unidentified and the sex of the remaining two unidentified individuals likely being male, the results were highly consistent among methods. The longest recorded pairwise movement was estimated at 151 km and involved a bear initially captured in the STAR‐P region in 2017, which was subsequently recaptured in the Rila Mountains of the Rhodope (RHO) region in later years. This individual was male, suggesting a possible case of natal dispersal. Male‐mediated gene flow is in accordance with observations in the EB and other bear populations (Straka et al. 2012; Frosch et al. 2014; Pylidis et al. 2021) with dispersal more likely to be related to population density or resource availability (Støen et al. 2006). The longest recorded female movement was 32 km. Additionally, one female bear detected in the RHO population was genetically assigned to the STAR‐P cluster (*Q* ≥ 0.8). In expanding populations, female bears have been shown to disperse over large distances (Jerina and Adamič 2008). However, in Bulgaria, where drive hunting for wild boar is still legally practiced, female bears have been observed travelling up to 15 km in a single day due to human disturbance (Valchev, observation of a GPS‐GSM collared bear), so instantaneous movements due to human disturbance cannot be ruled out.

Natural long‐distance movements would be good news for the persistence and functional connectivity of bears and the ecosystem of the Balkans. The Balkans are a genetic biodiversity hotspot (Gömöri et al. 2020) with high endemism, and brown bears are highly mobile seed dispersers (Lalleroni et al. 2017; García‐Rodríguez et al. 2021) and have a positive impact on biodiversity through cascade effects (Zyśk‐Gorczyńska et al. 2014; Grinath et al. 2015). At the same time, as bear populations expand and their individual activity is increasing near human settlements, measures need to be taken to minimise conflict with humans.

### 4.3 Asymmetrical Gene Flow

During population recovery, asymmetric gene flow is often seen in metapopulations (Vuilleumier and Possingham 2006), for example, in black bears (Malaney et al. 2018). In the Carpathians, asymmetric gene flow could occur during expansions from more abundant populations into recovering or peripheral patches (Matosiuk et al. 2019). By utilising a modest number of individuals from the Dinaric‐Pindos population, we were able to confirm the functional connectivity of the two main Balkan population hubs. Bayesian migration analyses revealed the pairwise functional connectivity between all groups, apart from S‐VLAH with RHO and STAR‐P with SHAR. STAR‐P and RHO population had a large degree of local ancestry, consistent with their relative geographical isolation. This could indicate that these populations have not reached carrying capacity. Migration between RHO and SHAR was strongly asymmetrical, with the prevailing direction from RHO to SHAR. Whether the asymmetrical gene flow we observed reflects a higher population density in source regions is an interesting hypothesis, but it is difficult to evaluate with the current data. Asymmetry aside, the observed connectivity is a positive element, as even one migrant per generation can rescue populations from the deleterious effects of genetic drift (Sagar et al. 2021).

### 4.4 Levels of Autosomal Variation

The genetic diversity of Eastern Balkan in our study was close to the reported values from other studies in the region (*H*_o_ = 0.64, Frosch et al. 2014; *H*_o_ = 0.71; Pylidis et al. 2021) and similar to other European populations (Swenson et al. 2011; Skrbinšek et al. 2012). The positive *F*‐values could suggest a degree of non‐random mating within these areas, but besides Wahlund effects, the technical challenges of working with non‐invasively collected hair and faecal samples could explain the lower‐than‐expected heterozygosity within clusters.

From BOTTLENECK, we detected signals of bottlenecks in all studied populations, and values of the G‐W statistics were below 0.5, suggesting past loss of alleles and a likely reduction in population size. We only estimated effective population sizes for EB, as we had larger sample sizes for this region. Both clusters were found below the *N*_e_ of 500 criterion, which has been suggested as the threshold for the long‐term viability of a population (Allendorf et al. 2012). In addition, the value for STAR‐P was found below the *N*_e_ = 50 criterion that is considered as the minimum which prevents a population from inbreeding. STAR‐P appears geographically as an island population, but gene flow from Rhodope, from the west and potentially from the Carpathian population in the north, may partially explain the moderately high heterozygosity despite its low Ne. The low *N*_e_ should be a reason for concern, especially as human‐wildlife conflict increases and illegal killing of bears and population fragmentation remain issues. The Balkans are recognised as a biodiversity hotspot for mammals, characterised by high species diversity and notable endemism, especially in mountainous regions such as the southern Dinarides and the Pindos Mt. range (Griffiths et al. 2004). This diversity is the result of the region's mountainous terrain, a diverse mosaic of habitats, and relatively high environmental stability over long periods of time, which have promoted both isolation and local adaptation among mammalian populations, enabling the diversification of lineages and simultaneously fostering the long‐term survival of diverse species, resulting in the emergence of endemism and subspecies with adaptive diversity, especially in areas with limited dispersal. This is supported by studies showing extensive within‐species genetic structuring and the presence of cryptic evolutionary lineages in carnivores, vertebrates and plants (Bazzicalupo et al. 2023; Horníková et al. 2021; Psonis et al. 2018; Španiel and Rešetnik 2022). This diversity underpins the region's role as a critical reservoir of genetic resources for European fauna, with important implications for conservation under changing climatic conditions.

### 4.5 Phylogeographic Signals

We detected the presence of two major mitochondrial lineages in the Eastern Balkan distribution which have been reported previously (Frosch et al. 2014; Pylidis et al. 2021). The MJ network depicts lineage 3a comprising Northern Scandinavia, North‐Eastern Europe, European Russia and the Balkans and lineage 1 found in Southern Europe. The Eastern lineage was found predominantly present in the Rhodope population in accordance with the findings of Frosch et al. (2014). Our neutrality test results for mtDNA control region sequences are consistent with previous evidence of population expansion following retractions around the last glacial maximum (Davison et al. 2011). In‐depth phylogeographic analyses are beyond the focus of this research and certainly require a larger dataset, but a phylogeographic contact zone in the area explains the detected high mtDNA genetic diversity and underpins the importance for conservation of admixture and reassortment of alleles in this gene pool, as argued by vonHoldt et al. (2018) for between‐species admixtures.

### 4.6 Outlook

Although our study detects signatures of population structuring in the central and south Balkans, our findings remain undesirably regional in scope, covering only a fraction of the species' distribution in the Balkans. Given that the populations in the Sharr and Stari Vlah mountains are ecologically and genetically connected to the broader populations of the central and southern Balkans, they cannot be considered in isolation. We therefore emphasise the importance of conducting a comprehensive study with hierarchical population structure analysis across the entirety of the brown bear distribution in the Balkans. This would allow us to accurately delineate the level of genetic structure, the detection of tentative distinct management units, and the development of conservation strategies across administrative and county borders (Liu et al. 2020). The formation of distinct evolutionary or management units is driven by a combination of historical isolation, gene flow, local adaptation and the ongoing environmental and climatic challenges. Nevertheless, even at this moderate scale and sampling, our project provided an opportunity for collaboration across geopolitical boundaries, which have often been a barrier for both humans and wildlife. Our work highlights the importance of conducting studies for large carnivore populations across borders and at an appropriate biological scale. Only by addressing population dynamics at appropriate scales can we ensure their long‐term viability in this ecologically and politically complex region.

## Author Contributions

**Charilaos Pylidis:** conceptualization (lead), data curation (equal), formal analysis (equal), funding acquisition (lead), investigation (equal), methodology (equal), project administration (lead), resources (lead), validation (equal), visualization (lead), writing – original draft (lead), writing – review and editing (lead). **Ivana Matic:** formal analysis (equal), investigation (supporting), validation (supporting), visualization (supporting), writing – original draft (equal). **Mihajla Djan:** formal analysis (equal), investigation (equal), writing – original draft (equal), writing – review and editing (equal). **Kostadin Valchev:** investigation (equal), project administration (supporting). **Duško Ćirović:** investigation (supporting), writing – original draft (supporting). **Simeon Arangelov:** investigation (supporting). **Sam Walker:** formal analysis (equal), investigation (equal), validation (supporting), writing – original draft (supporting). **Naroa Sarasua:** data curation (equal), investigation (supporting). **Veronika Mrazek:** investigation (supporting). **Ali E. Basuony:** formal analysis (supporting). **Michael W. Bruford:** conceptualization (equal), project administration (supporting), supervision (equal). **Koen Cuyten:** funding acquisition (lead), resources (lead). **Frank Hailer:** conceptualization (equal), data curation (equal), formal analysis (equal), funding acquisition (supporting), investigation (equal), methodology (equal), project administration (equal), resources (equal), supervision (lead), validation (equal), visualization (equal), writing – original draft (supporting), writing – review and editing (equal).

## Funding

This work was supported by Cardiff University School of Biosciences and Bears in Mind.

## Conflicts of Interest

The authors declare no conflicts of interest.

## Supporting information


**Appendix S1:** ece372691‐sup‐0001‐AppendixS1.docx.

**Appendix S2:** ece372691‐sup‐0002‐AppendixS2.xlsx.

## Data Availability

Mitochondrial sequences have been submitted to Genbank (accession numbers: PV980309–PV980316). Appendix S2 contains all genotype data, metadata and sequences of the mtDNA data. Other data or information is available from the corresponding authors upon reasonable request.
