# Supplementary material for: Dispersal Beyond Mountains and Borders: Asymmetrical Gene Flow Helps Maintain Brown Bear Metapopulation Connectivity on the Balkan Peninsula
Source: Ecol Evol. 2025 Dec 18;15(12):e72691. doi: 10.1002/ece3.72691 (PMC12712544; doi:10.1002/ece3.72691)
Supplement: Supplementary file 1 — Appendix S1: ece372691‐sup‐0001‐AppendixS1.docx. [file ECE3-15-e72691-s002.docx]

### Supplementary Data S1.

### *Details of the ten microsatellite loci used in the present study*

| Primer | Multiplex | Sequence | Annealing T (°C) | Dye | µL of each primer in  15 µL reaction | Allele range (bp)# |
| --- | --- | --- | --- | --- | --- | --- |
| G1A | 1 | F: ACCCTGCATACTCTCCTCTGATG | 58 | 6-FAM | 0.5 | 177-195 |
|  |  | R: GCACTGTCCTTGCGTAGAAGTGAC |  |  |  |  |
| Mu05 | 1 | F: ATGTGGATACAGTGGAATAGACC | 58 | NED | 0.5 | 109-133 |
|  |  | R: GTTTCTTGTGACATGAACTGAAACTTGTTAT |  |  |  |  |
| Mu51 | 1 | F: AGCCAGAATCCTAAGAGACCT | 60 | HEX | 0.5 | 105-129 |
|  |  | R: AAAGAGAAGGGACAGGAGGTA |  |  |  |  |
| G10L | 1 | F: ACTGATTTTATTCACATTTCCC | 60 | HEX | 0.5 | 141-161 |
|  |  | R: GATACAGAAACCTACCCATGCG |  |  |  |  |
| G10J | 1 | F: GATCAGATATTTTCAGCTTT | 52 | 6-FAM | 0.5 | 73-103 |
|  |  | R: AACCCCTCACACTCCACTTC |  |  |  |  |
| G10P | 2 | F: AGTTTTACATAGGAGGAAGAA | 58 | 6-FAM | 0.6 | 141-173 |
|  |  | R: TCATGTGGGGAAATACTCTGAA |  |  |  |  |
| G10C | 2 | F: AAAGCAGAAGGCCTTGATTTCCTG | 58 | NED | 0.2 | 87-109 |
|  |  | R: GGGACATAAACACCGAGACAGC |  |  |  |  |
| Mu23 | 2 | F: GCCTGTGTGCTATTTTATCC | 60 | HEX | 0.6 | 136-156 |
|  |  | R: TAGACCACCAAGGCATCAG |  |  |  |  |
| Mu50 | 2 | F: GTCTCTGTCATTTCCCCATC | 60 | 6-FAM | 0.5 | 76-102 |
|  |  | R: AACCTGGAACAAAAATTAACAC |  |  |  |  |
| Mu59 | 2 | F: GCTCCTTTGGGACATTGTAA | 60 | HEX | 0.6 | 90-122 |
|  |  | R: TGACTGTCACCAGCAGGAG |  |  |  |  |

# as given in the original primer note

**References**

Paetkau, D., & Strobeck, C. (1994). Microsatellite analysis of genetic variation in black bear populations. *Molecular Ecology*, 3(4), 489-495.

Paetkau, D., Shields, G.F., & Strobeck, C. (1995). Gene flow between insular, coastal and interior populations of brown bears in Alaska. *Molecular Ecology*, 4(4), 403-410.

Taberlet, P., Waits, L.P., & Luikart, G. (1997). Noninvasive genetic sampling: look before you leap. *Trends in Ecology & Evolution*, 12(9), 363-364.

### Supplementary Data S2.

### *Mitochondrial DNA methods*

Each reaction was conducted in a 10 µL volume composed of: 5 µL of Qiagen Multiplex PCR Master Mix (Qiagen, Manchester, UK), 2.0 µL of water, 0.5 µL of forward primer, 0.5 µL of reverse primer and 2 µL of DNA. For the PCR conditions, the two primer pairs were run in separate tubes but at the same conditions which were 95°C for 15 minutes, then 40 cycles of 94°C for 30 seconds, 59°C for 90 seconds and 72°C for 60 seconds. This was followed by a final extension at 72°C for 15 minutes, with the PCR products cooled down to 14°C before being stored in a fridge until further analysis. To identify successful amplifications, 3 µL of PCR products were run on 1% agarose gels for 30 minutes, stained with GelRed (Biotinum) or SYBRSafe (Thermo Fisher).

Mitochondrial PCR products were then diluted for Sanger sequencing. 1.5 µL of PCR products were diluted with 13.5 µL of water for samples with good amplification (strong bands on agarose). For samples with weaker bands, 3 µL of PCR products were diluted with 12 µL of water . PCR products were sequenced in both directions , adding 2 µL of forward or reverse primer, respectively, to each reaction. Sanger dideoxy sequencing was conducted by TubeSeq service by Eurofins (UK).

Trimming of sequencing reads, sequence edits and the production of sequence contigs were performed using the software Geneious Prime 2023.0.4 (Biomatters 2020). Sequence contigs were generated by combining the forward and reverse reads for each individual separately, using the ‘Map to reference’ function. The sequences were mapped to a complete clade 3a brown bear mitochondrial genome downloaded from GenBank (see Supplementary Information B). This fully annotated sequence was trimmed to only include the mitochondrial control region and tRNA-pro gene targeted by the chosen primers. All obtained sequence data was then trimmed, removing lower quality sections and retaining only sequence inside (i.e., excluding) the primer binding regions All sequences were then aligned with a range of reference sequences representing the main brown and polar bear clades, as well as haplotypes found in the Balkan study area in previous studies (see Supplementary Information, B).

**References**

Biomatters. 2020. Geneious - Bioinformatics Software for Sequence Data Analysis. *Geneious*.

### Supplementary Data S3.

### *Sexing Methods*

For molecular sexing of the non-invasively collected samples, we followed the method by Bidon et al. (2013). Two Y-chromosome specific fragments, *SMCY* and *318.2,* along with one X-specific fragment *ZFX,* were amplified in a single multiplex. The multiplex mix for the sexing PCR was conducted in 10 µL reaction volumes composed of 5 µL Qiagen Multiplex PCR Master Mix (Qiagen, Manchester, UK, ID: 206143), 1 µL of SMCY (10 µM), 1 µl of ZFX (10 µM), 0.8 µL of 318.2 (10 µM) and 0.2 µL of water (Bidon et al. 2013). For individuals that gave inconclusive results, this mix was later altered to include bovine serum albumin (BSA) and was as follows: 5 µL Qiagen Multiplex PCR Master Mix (Qiagen, Manchester, UK, ID: 206143), 2.3 µL water, 0.3 µL SMCY (10 µM), 0.3 µL ZFX (10 µM), 0.25 µL 318.2 (10 µM) and 0.05 µL of BSA (10 µg/µl). In the PCR machine (GeneAmp PCR Systems 9700), all samples underwent touchdown PCR at the following conditions: 95°C for 15 minutes, then 10 cycles of 94°C for 30 seconds, 69°C (-1°C per cycle) for 25 seconds and 72°C for 75 seconds. This was followed by 30 cycles of 94°C for 30 seconds, 59°C for 25 seconds and 72°C for 75 seconds. For 15 minutes, the samples were held at 72°C and then cooled to 14°C. For the altered method including BSA, a different PCR program was used, with the touchdown extended to cool by 0.5°C per cycle for 20 cycles. PCR products were run on 3% agarose gel for 90 minutes, stained with GenRed and bands visualised under UV light. All samples were sexed a minimum of two times, with repeats conducted independently from each other in separate PCR mixes but using the same DNA extracts. References

**References**

Bidon, T. et al. 2013. A sensitive and specific multiplex PCR approach for sex identification of ursine and tremarctine bears suitable for non-invasive samples. *Molecular Ecology Resources* 13(3). doi: 10.1111/1755-0998.120

### Supplementary Data S4.

### *PID analysis for the 10 loci used in this study*


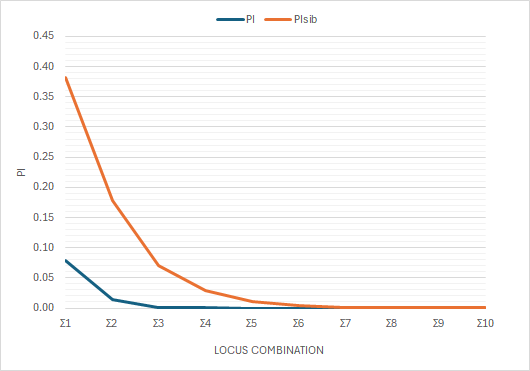


### Supplementary Data S5. The most probable number of clusters

#### S5.1 All DIN-PIN and EB profiles (dataset without relatives, n=82)

Arrow indicates best clustering for LnP(K)


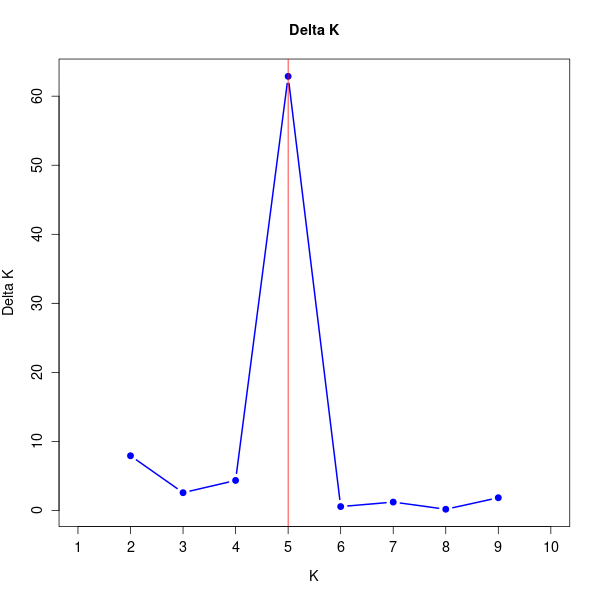

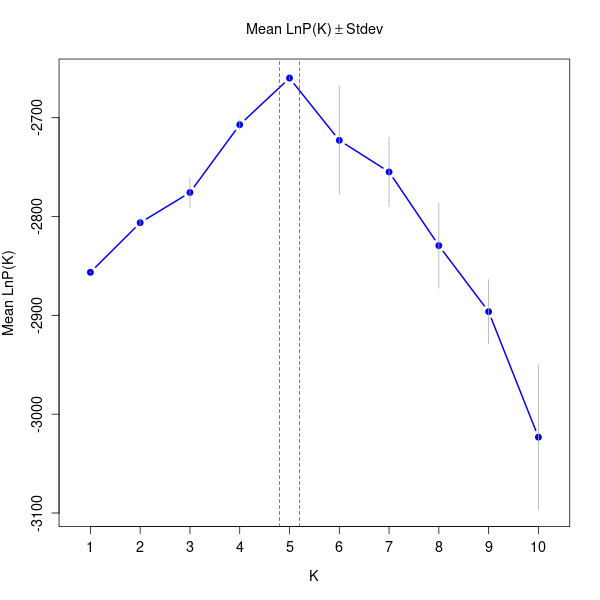


#### S5.2 Eastern Balkan bears only (n=62) relatives removed

Arrow indicates best clustering for LnP(K)


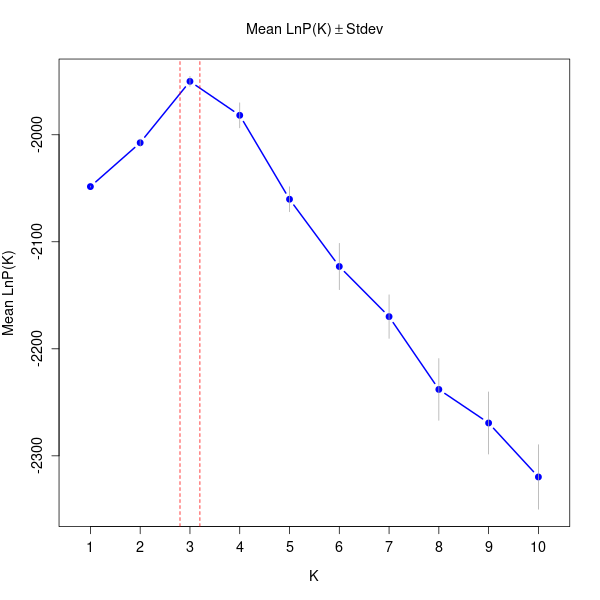

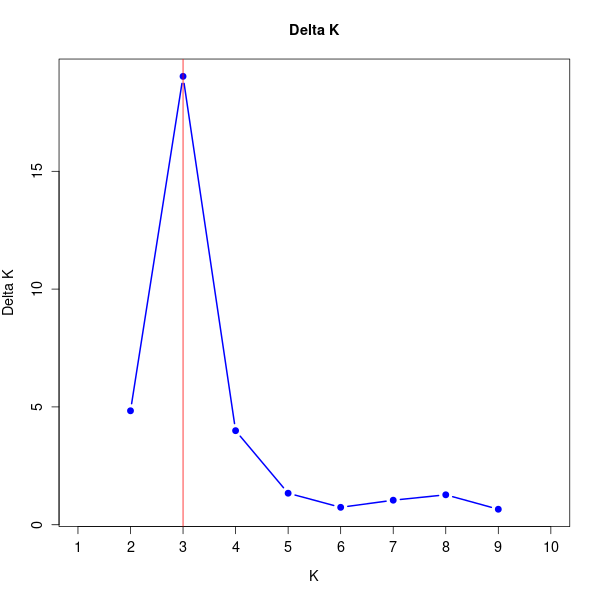


**K=2**


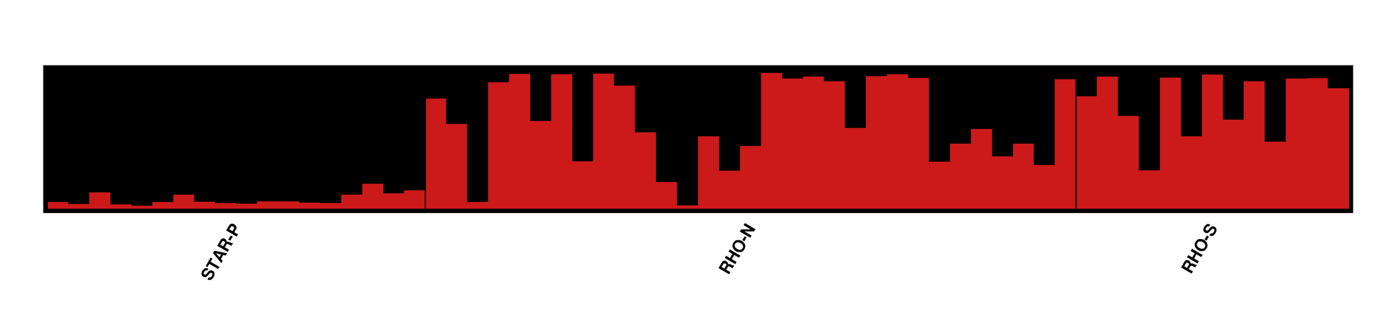


**K=3**


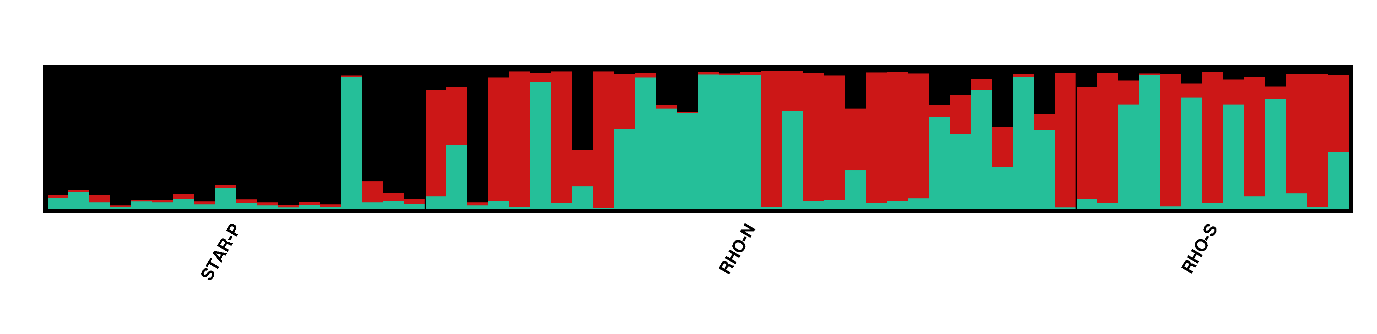


#### Supplementary Data S5.3 — Eastern Balkan bears only (n=96) / relatives present

Circled area and arrow indicate best clustering for LnP(K)


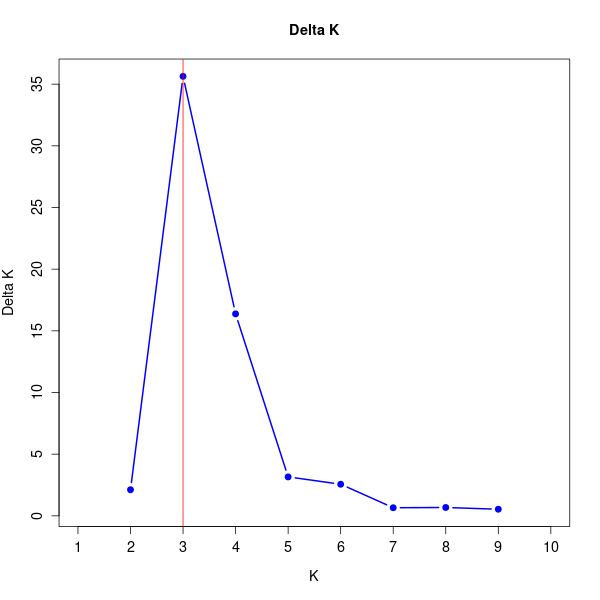

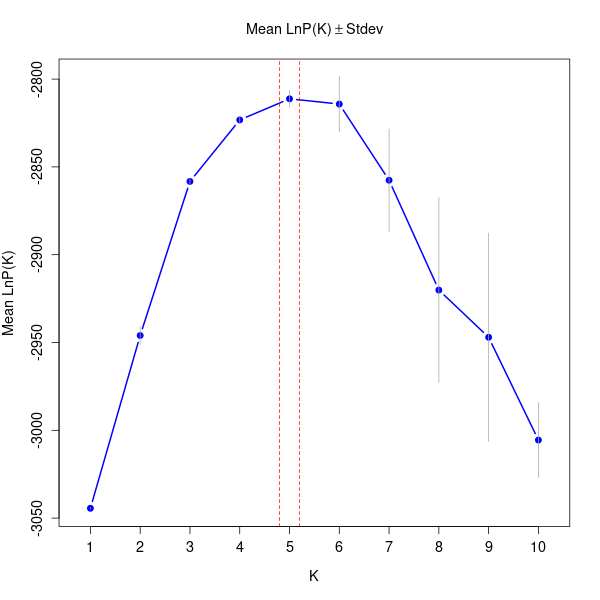


**K=3**


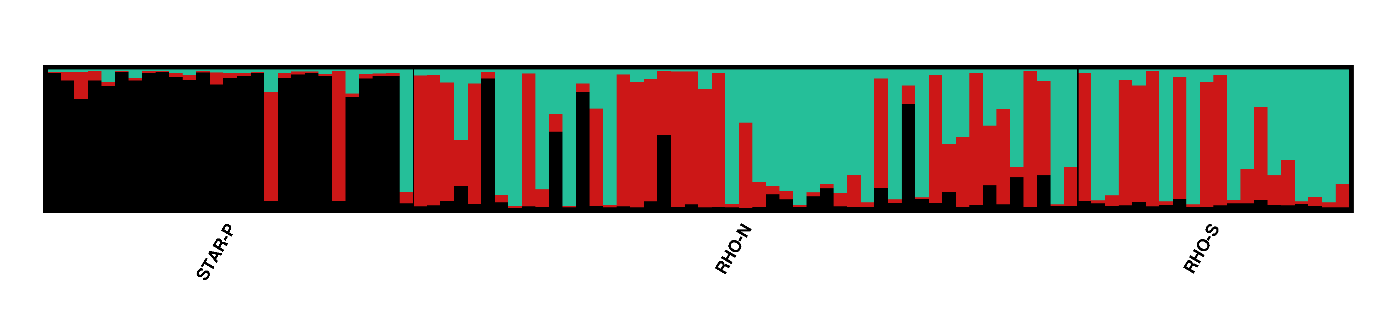


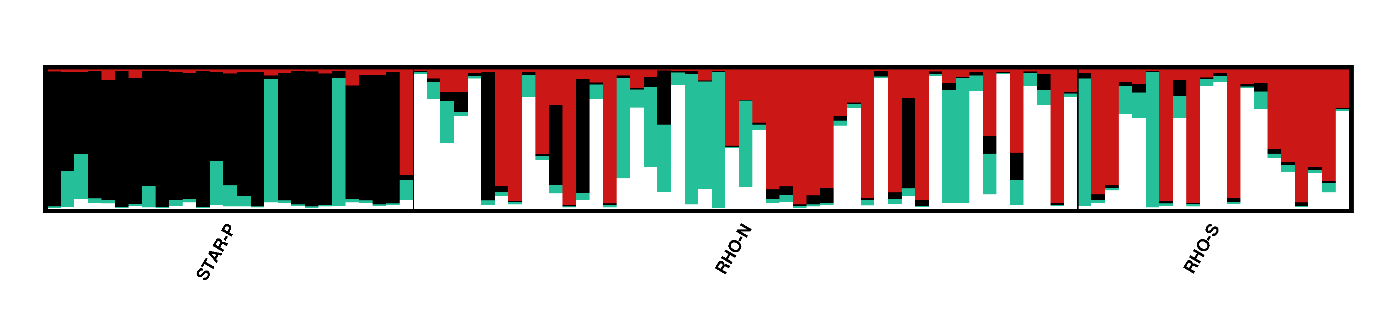
**K=4**

#### S5.4 All individual profiles from all four regions (relatives present in dataset) n=122

Arrow indicates best clustering for LnP(K)


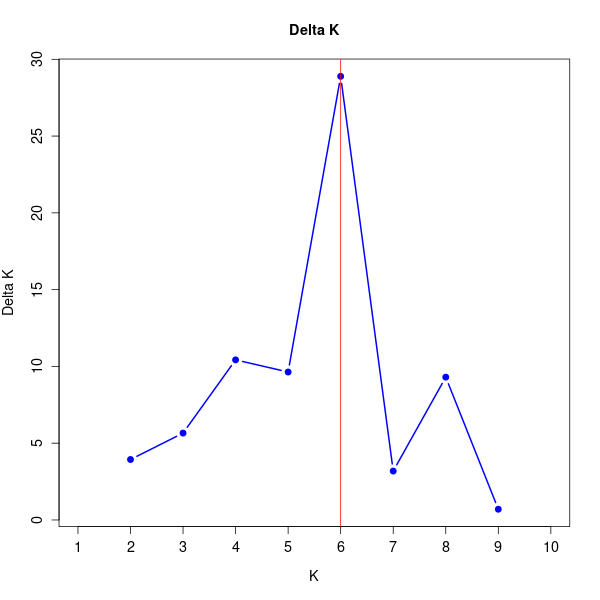

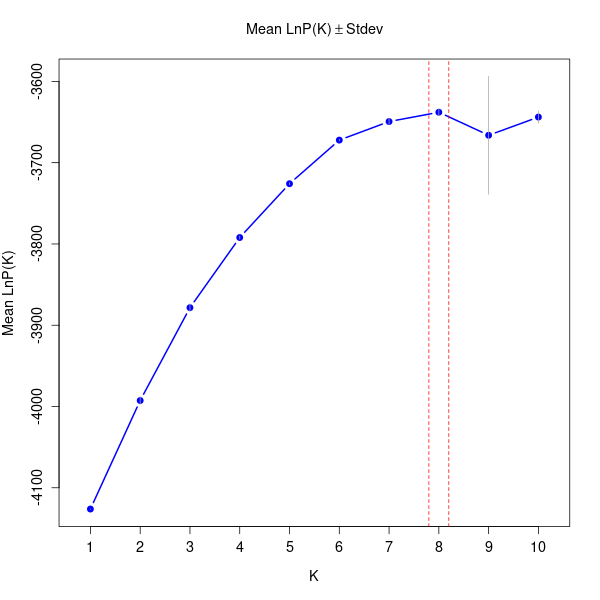


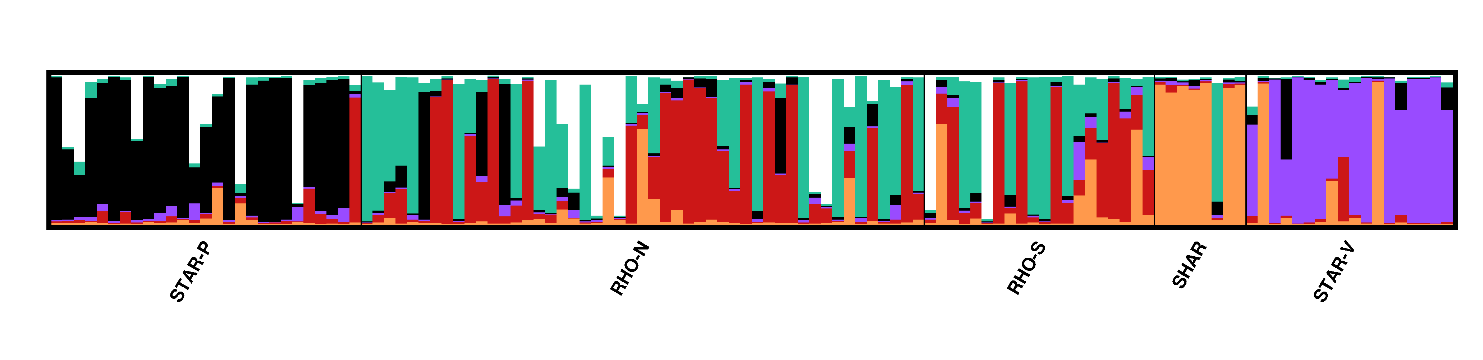
**K=6**

### Supplementany Data S6.

#### Bayesian estimation of unidirectional migration rates in BayesAss

Individuals: 122, Populations: 4, Loci: 10

MCMC Iterations=100000000

Burn-in=10000000

Sampling Interval=10000

Mixing parameters: (dM=0.35,dA=0.7,dF=0.7)

|  |  | 0-> Star-P |  | 1-> Rho |  | 2-> Shar |  | 3-> S-Vlah |
| --- | --- | --- | --- | --- | --- | --- | --- | --- |
| 0->Star-P | m[0][0]: | 0.9458  (0.0281) | m[0][1]: | 0.0289  (0.0234) | m[0][2]: | 0.0122  (0.0119) | m[0][3]: | 0.0131  (0.0128) |
| 1->Rho | m[1][0]: | 0.0261  (0.0159) | m[1][1]: | 0.9633  (0.0173) | m[1][2]: | 0.0055  (0.0055) | m[1][3]: | 0.0050  (0.0049) |
| 2->Shar | m[2][0]: | 0.0351  (0.0321) | m[2][1]: | 0.0884  (0.0537) | m[2][2]: | 0.8460  (0.0608) | m[2][3]: | 0.0306  (0.0279) |
| 3-> S-Vlah | m[3][0]: | 0.0369  (0.0261) | m[3][1]: | 0.0186  (0.0178) | m[3][2]: | 0.0550  (0.0280) | m[3][3]: | 0.8895  (0.0370) |

Meirman’s deviance: 7296.723448

### Supplementany Data S7.

#### S7.1 Bottleneck results

| **Parameters** | **T.P.M.: Variance=12.00 Probability=95.00%** | |
| --- | --- | --- |
| Population | p_W_2t_IAM | p_W_2t_TPM |
| STAR-P | 0.006836*** | 0.695313 |
| RHO | 0.00488*** | 0.27539 |
| DINcomb | 0.000977*** | 0.160156 |
| Parameters | T.P.M.: Variance=20.00 Probability=95.00% | |
| Population | p_W_2t_IAM | p_W_2t_TPM |
| STAR-P | 0.006836*** | 0.695313 |
| RHO | 0.00488*** | 0.37500 |
| DINcomb | 0.000977*** | 0.032227* |
| Parameters | Variance=12.00 Probability=70.00% | |
| Population | p_W_2t_IAM | p_W_2t_TPM |
| STAR-P | 0.004883*** | 0.232422 |
| RHO | 0.00488*** | 0.92188 |
| DINcomb | 0.000977*** | 0.001953*** |
| Parameters | Variance=20.00 Probability=70.00% | |
| Population | p_W_2t_IAM | p_W_2t_TPM |
| STAR-P | 0.006836*** | 0.193359 |
| RHO | 0.00488*** | 0.69531 |
| DINcomb | 0.000977*** | 0.000977*** |

#### S7.2 Garza-Williamson index

------------------------------------------------------------------------------

**Locus STAR-P RHO SHAR S-VLAH Mean s.d.**

------------------------------------------------------------------------------

1 0.20690 0.31250 0.25926 0.25926 0.25948 0.03734

2 0.33333 0.35714 0.37500 0.33333 0.34970 0.01754

3 0.55556 0.36364 0.45455 0.46154 0.45882 0.06790

4 0.44444 0.41667 0.22222 0.25000 0.33333 0.09821

5 0.43750 0.36000 0.40000 0.42857 0.40652 0.03022

6 0.22581 0.23529 0.50000 0.24138 0.30062 0.11525

7 0.21429 0.32000 0.31579 0.32143 0.29288 0.04542

8 0.22222 0.27778 0.16667 0.22222 0.22222 0.03928

9 0.31250 0.42105 0.21429 0.42857 0.34410 0.08790

10 0.33333 0.34783 0.31579 0.44444 0.36035 0.04986

------------------------------------------------------------------------------

Mean 0.32859 0.34119 0.32236 0.33907 0.33280 0.00769

s.d. 0.11263 0.05453 0.10385 0.08942 0.09011 0.02215

------------------------------------------------------------------------------

### Supplementary Data S8.

### *List of new haplotype sequences*

Eastern Balkan population

H3-EB7     PV980309

CCAGCACCCAAAGCTAATGTTCTATTTAAACTATTCCCTGGTACATACTACTATTTTACTCTGTGTCCTATTTATTTCATATATACCATCTTATGTACTGTGCCATCACAGTATGTGTATATCGTGCATTAATGGCGTGCCCCATGCATATAAGCATGTACATATTATGCTTGGCCTTACATGAGGACTTACGTTCCGAAAGTTTGTTTCAGGCGTATAGTCTGTAAGCATGTATTTCACTTAGTCCGGGAGCTTAGTCACCAGGCCTCGAGAAACCAGCAATCCTTGCGAGTACGTGTACCTCTTCTCGCTCCGGGCCCATGAAGTGTGGGGGTTTCTATGCTGAAACTATACCTGGCATCTGGTTCTTACCTCAGGGCCATGAT

H5-EB6     PV980310

TCAGCACCCAAAGCTAATGTTCTATTTAAACTATTCCCTGGTACATACTACTATTTTACCCCGTGTCCTATTTATTTCATATATACCATCTTATGTACTGTACCATCACAGTATGTGTATATCGTGCATTAATGGCGTGCCCCATGCATATAAGCATGTACATATTGTGCTTGGTCTTACATGAGGACTTACATTCCGAAAGTTTGTTTCAGGTGTATAGTCTGTAAGCATGTATTTCACTTAGTCCAGGAGCTTAGTCACCAGGCCTCGAGAAACCAGCAATCCTTGCGAGTACGTGTACCTCTTCTCGCTCCGGGCCCATGAAGTGTGGGGGTTTCTATGTTGAAACTATACCTGGCATCTGGTTCTTACCTCAGGGCCATGACAGC

H6-EB8     PV980311

AAAGCTAATGTTCTATTTAAACTATTCCCTGGTACATACTACTATTTTACCCCATGTCTTATTCATTTCATATATACCATCTTATGTACTGTACCATCGCAGTATGTGTATATCGTGCATTAATGGCGTGCCCCATGCATATAAGCATGTACATATTACGTTTGGTCTTACATAAGGACTTACGTTCCGAAAGCTTATTTCAGGCGTATGGTCTGTAAGCATGTATTTCACTTAGTCCGGGAGCTTGATCACCAGGCCTCGAGAAACCAGCAACCCTTGCGAGTACGTGTACCTCTTCTCGCTCCGGGCCCATGGGATGTGGGGGTTTCTATGTTGAAACTATACCTGGCATCTGGTTCTTACTTCAGGGCCATGATAGCTCTAGATTCCAATCCTACTAACC

H9-EB11   PV980312

CTATTTAAACTATTCCCTGGTACATACTACTATTTTACTCTGTGTCCTATTTATTTCATATATACCATCTTATGTACTGTGCCATCACAGTATGTGTATATCGTGCATTAATGGCGTGCCCCATGCATATAAGCATGTACATATTATGCTTGGTCTTACATGAGGACTTACGTTCCGAAAGTTTGTTTCAGGCGTATAGTCTGTAAGCATGTATTTCACTTAGTCCGGGAGCTTAATCACCAGGCCTCGAGAAACCAGCAATCCTTGCGAGTACGTGTACCTCTTCTCGCTCCGGGCCCATGAAGTGTGGGGGTTTCTATGCTGAAACTATACCTGGCATCTGGTTCTTACCTCAGGGCCATGACAGCTCTAGATTCCAATCCTACTAACC

Dinaric-Pindos population

H11-D2     PV980313

AAAGCTAATGTTCTATTTAAACTATTCCCTGGCACATACTACTATTTTACTCCGTGTCCTATTTATTTCATATATACCATCCTATGTACTGTACCATCACAGTATGTGTATATCGTGCATTAATGGTGTGCCCCATGCATATAAGCATGTACATACTGTGCTTGGTCTTACATGAGGACTTACGTTTCGAAAGTTTATTTCAGGCGTATAGTCTGTAAGCATGTATTTCACTTAGTCCGGGAGCTTAGTCACCAGGCCTCGAGAAACCAGCAACCCTTGCGAGTACGTGTACCTCTTCTCGCTCCGGGCCCATGAAGTGTGGGGGTTTCTATGTTGAAACTATACCTGGCATCTGGTTCTTACCTCAGGGCCATGATAGCTCTAGATTCCAATCCTACTAACC

H12-D5     PV980314

TCAGCACCCAAAGCTAATGTTCTATTTAAACTATTCCCTGGCACATACTACTATTTTACTCCGTGTCCTATTTATTTCATATATACCATCCTATGTACTGTACCATCACAGTATGTGTATATCGTGCATTAATGGTGTGCCCCATGCATATAAGCATGTACATACTGTGCTTGGTCTTACATGAGGACTTACGTTCCGAAAGTTTATTTCAGGCGTATAGTCTGTAAGCATGTATTTCACTTAGTCCGGGAGCTTAGTCACCAGGCCTCGAGAAACCAGCAACCCTTGCGAGTACGTGTACCTCTTCTCGCTCCGGGCCCATGAAGTGTGGGGGTTTCTATGTTGAAACTATACCTGGCATCTGGTTCTTACCTCAGGGCCATGATAGC

H13-D3     PV980315

AAAGCTAATGTTCTATTTAAACTATTCCCTGGTACATACTACTATTTTACTCCGTGTCCTATTTATTTCATATATACCATCTTATGTACTGTGCCATCACAGTATGTGTATATCGTGCATTAATGGCGTGCCCCATGCATATAAGCATGTACATATTGTGCTTGGTTTTACATGAGGACTTACGTTCCGAAAGCTTGTTTCAGGTGTATAGTCTGTAAGCATGTATTTCACTTAGTCCGGGAGCTTAGTCACCAGGCCTCGAGAAACCAGCAATCCTTGCGAGTACGTGTACCTCTTCTCGCTCCGGGCCCATGAAGTGTGGGGGTTTCTATGTTGAAACTATACCTGGCATCTGGTTCTTACCTCAGGGCCATGACAGCTCTAGATTCCAATCCTACTAACC

H14-D4     PV980316

CCTGGTACATACTACTATTTTACTCCGTGTCCTATTTATTTCATATATACCATCTTATGTACTGTGCCATCACAGTATGTGTATATCGTGCATTAATGGCGTGCCCCATGCATATAAGCATGTACATATTGTGCTTGGTTTTACATGAGGACTTACGTTCCGAAAGCTTGTTTCAGGCGTATAGTCTGTAAGCATGTATTTCACTTAGTCCGGGAGCTTAGTCACCAGGCCTCGAGAAACCAGCAATCCTTGCGAGTACGTGTACCTCTTCTCGCTCCGGGCCCATGAAGTGTGGGGGTTTCTATGTTGAAACTATACCTGGCATCTGGTTCTTACCTCAGGGCCATGACAGCTCTAGATTCCAATCCTACTAACC
